# Role of Exercise in Vascular Function and Inflammatory Profile in Age-Related Obesity

**DOI:** 10.1155/2018/7134235

**Published:** 2018-10-28

**Authors:** Anna Pedrinolla, Massimo Venturelli, Emine Kirmizi, Federica Moschetta, Monica Zardini, Doriana Rudi, Elisabetta Bacchi, Federico Schena, Paolo Moghetti, Massimo Lanza

**Affiliations:** ^1^Department of Neuroscience, Biomedicine and Movement Science, University of Verona, Verona, Italy; ^2^Division of Endocrinology, Diabetes and Metabolism, Department of Medicine, University of Verona and Hospital Trust of Verona, Verona, Italy; ^3^Department of Internal Medicine, University of Utah, USA; ^4^Department of Physiologu, Faculty of Medicine, Uludag University, Eskisehir, Turkey

## Abstract

In western countries, aging is often accompanied by obesity and age-related obesity is characterized by vascular dysfunction and a low-grade inflammatory profile. Exercise is a nonpharmacological strategy able to decrease the development and incidence of risk factors for several health-threatening diseases. Nonetheless, its long-term effect on vascular function and inflammation in age-related obesity is still unclear. The aim of this study was to investigate the effect of regular, supervised exercise on inflammatory profile and vascular function in age-related obesity. We also hypothesized that vascular function and inflammatory profile would have been correlated in overweight and obese individuals. Thirty normal weight (NW; 70 ± 5 years, 23.9 ± 2.6 BMI) and forty overweight and obese elderly (OW&OB; 69 ± 5 years, 30.1 ± 2.3 BMI) regularly taking part in a structured, supervised exercise program were enrolled in the study and evaluated for vascular function (flow-mediated dilation; FMD) and inflammatory profile (plasma CRP, IL-1*β*, IL-1ra, IL-6, IL-8, IL-10, TNF-*α*, and MCP-1). Although no differences between groups were found concerning performance and the weekly amount of physical activity, the OW&OB group compared with the NW group demonstrated higher systolic and diastolic blood pressure (+10%, *p* = 0.001; +9%, *p* = 0.005, respectively); lower FMD% (−36%, *p* < 0.001) and FMD/shear rate (−40%, *p* = 0.001); and higher levels of CRP (+33%, *p* = 0.005), IL-6 (+36%, *p* = 0.048), MCP-1 (+17%, *p* = 0.004), and TNF-*α* (+16%, *p* = 0.031). No correlations between vascular function and inflammation were found in OW&OB or NW. Although exercising regularly, overweight and obese elderly exhibited poorer vascular function and higher proinflammatory markers compared with the leaner group. These results support the idea that exercise alone cannot counteract the negative effect of adiposity on vascular function and inflammatory profile in elderly individuals and these two processes are not necessarily related.

## 1. Introduction

Aging, an inevitable and unstoppable process, is a well-documented risk factor for cardiovascular and atherosclerotic disease and it is associated with a progressive decline in endothelium-dependent vasodilation in both resistance and conduit arteries [1, 2]. These alterations are primarily related to a worsening of mitochondrial function, increased inflammatory profile, augmented free radical production, and a gradual loss of antioxidant capacity [2, 3]. Altogether, these mechanisms lead to an impairment of nitric oxide (NO) bioavailability [1–3], with reduced endothelium-dependent vasodilation, serving as a risk factor for diabetes, hypertension, dyslipidemia, and several forms of cancer [4]. Obesity is another condition known to be characterized by vascular dysfunction and, differently from aging, is a reversible state. Indeed, increased body fat is associated with increased inflammatory profile, as well as with increased reactive oxygen species (ROS) production and impaired NO-mediated endothelium-dependent vasodilation [5, 6]. Older adults represent the faster growing population in Europe and all over the world, and the prevalence of obesity among this population is about 20–30% [7]. Indeed, aging is also characterized by increased fat mass and loss of fat-free mass, with a concomitant increase of overall adiposity stored primarily as triglycerides in subcutaneous and visceral adipose tissue, as well as in nonadipose tissues such as skeletal muscles and liver [8]. Consequently, the aging process itself and the age-dependent accumulation of adipose tissue contribute both to the onset and development of NO-mediated vascular dysfunction with a detrimental effect on the incidence of cardiometabolic diseases such as hypertension, dyslipidemia, and diabetes, as well as the development of cancer [5, 9–11]. However, evidence showing the relation between higher inflammatory profile and lower vascular function in obese elderly individuals is lacking and specific studies are needed.

Recent evidence has shown that physical activity can improve the inflammatory profile and endothelium-dependent vasodilation in healthy individuals as well as in individuals with endothelial dysfunction associated with chronic conditions [1]. Physical training has beneficial effects on several cardiovascular risk factors such as dyslipidemia, hypertension, and diabetes, and can serve as an intervention to lower the risk of developing several health-threatening conditions such as cardiovascular chronic disease and cancer [2]. Studies on healthy and active aging have shown that in contrast to their sedentary counterparts, older men who performed regular aerobic exercise have largely preserved vascular function, lower low-grade inflammation, and reduced risk factors for several invalidating diseases [3, 4, 6, 12]. However, a recent study aimed at evaluating the effect of long-term physical activity intervention on vascular health and inflammatory biomarkers in the elderly did not provide further evidence for the beneficial effect of regular exercise in older adults [13]. Recently, some publications have emerged claiming that physical activity counteracts obesity as a risk factor [5, 10, 14]. Nevertheless, other studies have shown that regular physical activity protects against incident cardiovascular disease, but it does not eliminate the harmful effect of overweight and obesity [5, 11, 15–17]. Moreover, it is important to highlight that no studies have aimed at investigating the exercise-induced effects on vascular function and inflammatory profile in age-related obesity.

Consequently, due to contrasting results about the effect of regular exercise on inflammatory profile and vascular function in aging and obesity, and the lack of evidence about the effect of exercise on aging-induced obesity, the aim of this study was to investigate the effect of regular, supervised exercise on inflammatory profile and vascular function in nonobese and obese elderly who habitually take part in a supervised exercise program. The aim of this study was to investigate the effects of regular, supervised exercise on vascular function and inflammatory profile in obese and nonobese older individuals.

## 2. Methods

### 2.1. Subjects

The study population included 70 physically active older adults (30 females, 40 males, mean age 70 ± 5 years, 30 nonobese, 15 overweight, and 25 obese) (Table 1) who habitually took part in a structured, supervised exercise program. Subjects were included in the study in the absence of smoking history, atherosclerotic vascular disease, heart failure, and liver, renal, or inflammatory disease, and if they did not experience great body mass index (BMI) variation in the last 5 years (≤0.7 kg ∙ (m^2^)^−1^). None of the subjects was subjected to a caloric restriction diet at the time of the study. Subjects were informed about testing procedures, possible risks, and discomfort that might ensue and gave their written informed consent to participate in accordance with the Declaration of Helsinki, as part of a protocol approved by the Institutional Review Board of the Azienda Ospedaliera Universitaria Integrata of Verona, Italy (protocol # 788CESC).

One day preceding the tests, the subjects were prohibited from training and their normal diet was maintained. A vascular test and blood sampling were carried out on the same day. Room temperature was 22–24°C, and relative humidity was 50%. All the ultrasound studies were performed by a single experienced vascular sonographer who was unaware of the clinical and laboratory characteristics of the subjects. Height and weight were recorded by means of a scale and stadiometer (Seca, Hamburg, Germany), and body mass index (BMI) was calculated as weight (kg)/height (m)^2^.

The study was conducted in September 2017, right before the beginning of the annual exercise program (which starts in September and ends in July, having about a 4-week break in summer). The data were collected at the beginning of a new year of the exercise program from subjects who have been taking part in this program for about 5 years, 3 days a week, allowing us to consider these people as active and trained even after a break of a few weeks. Also, a battery of performance and functional tests including a 6-minute walking test (6MWT) was performed at the beginning of the exercise program in order to set the exercise goals. Briefly, 6MWT measures the maximum distance that a person can walk over 6 min and it is commonly used as an assessment of exercise capacity. The distance (m) covered in 6 min was recorded [18].

### 2.2. Blood Samples and Analysis

Venous blood samples were drawn from an antecubital vein and collected into EDTA tubes for analysis of inflammatory profiles. The samples were centrifuged for 10 minutes at +4°C with 2500 ×g. Plasma was kept at −80°C until analyzed for interelukine-1ra (IL1-ra; Chi, 4.67%; CV, 0.034%; DC = (0.10, 18011.00)), interleukin-1*β* (IL-1*β*; Chi, 6.86%; CV, 0.65%; DC = (0.057, 23660.71)), interleukin-6 (IL-6; Chi, 7.70%; CV, 0.36%; DC = (0.39, 47602.24)), interleukin-8 (IL-8; Chi, 2.15%; CV,0.21%; DC = (0.18, 41913.46)), interleukin-10 (IL-10; Chi, 7.29%; CV, 0.66%; DC = (0.36, 10173.63)), tumor necrosis factor-*α* (TNF-*α*; Chi, 7.02%; CV, 0.77%; DC = (0.28, 15643.48)), and monocyte chemoattractant protein-1 (MCP-1; Chi, 4.73%; CV, 0.68%; DC = (0.43, 21239.64)), which were determined by means of the commercially available MILLIPLEX multianalyte panel HCYTOMAG-60K-07 (Merck Millipore, Darmstadt, Germany) following the manufacturer's recommendation. C-reactive protein (CRP) was measured using an ELISA commercial kit, following the manufacturer's recommendation (Diagnostics Biochem Canada Inc. (DBC), London, Canada). We included both pro- and anti-inflammatory markers related with exercise as well as related with the development of risk factors for several health-threatening diseases.

### 2.3. Flow-Mediated Dilation (FMD) Test

The FMD test has been proposed to represent a functional bioassay for endothelium-derived NO bioavailability and vascular function in humans [19]. The FMD test was performed in a quiet room with participants in abstinence from alcohol, antioxidants (i.e., orange juice and beetroot juice), and caffeine for at least 12 h [20]. High-resolution ultrasound was used to image the brachial artery at rest and after 5 min of ischemia. All the FMD tests were performed with the participant in the supine position, with the right arm extended at an angle of ~90° from the torso. The brachial artery was imaged using the high-resolution ultrasound system LOGIQ-7 ultrasound Doppler system (General Electric Medical Systems, Milwaukee, WI, USA). The ultrasound Doppler system was equipped with a 12–14 MHz linear array transducer. The brachial artery was imaged 5–10 cm above the antecubital fossa in the longitudinal plan, and the diameter was determined at 90° angle along the central axis of the scanned area. When an optimal image was acquired, the position was maintained for the whole test and all scans were stored for later analysis. After baseline brachial artery imaging, a blood pressure cuff was placed around the forearm and inflated to 250 mmHg for 5 min. Brachial artery images and blood velocity were obtained continuously 30 s before and 2 min after cuff release [19]. The brachial artery images were analyzed by a blinded investigator by means of FloWave.US [21]. Arterial diameter was measured as the distance (mm) between the intima-lumen interfaces for the anterior and posteriors walls. Utilizing arterial diameter and the blood velocity, blood flow (BF) was calculated as follows:

(1) $$\begin{array}{c} BF\;\left(ml/\min\right)=blood\;velocity\cdot \Pi \cdot {\left(\frac{vessel\;diameter}{2}\right)}^{2}\cdot 60. \end{array}$$

The calculation of FMD as a percentage change uses the peak diameter in response to reactive hyperemia in relation to the baseline diameter and was calculated as follows:

(2) $$\begin{array}{c} FMD\left(\%\right)=\frac{\left(peak\;diameter-baseline\;diameter\right)}{baseline\;diameter}, \end{array}$$

and when multiplied by 100, FMD is expressed as a percentage of change in the vessel caliber [19].

Postcuff release shear rate was calculated using the following equation: shear rate (*s*^−1^) = 8*V*_mean_/vessel diameter. Cumulative shear rate (*s*^−1^∙*s*) and the reactive postcuff release (total blood flow over 2 min) were integrated using the trapezoidal rule and calculated as

(3) $$\begin{array}{c} \sum \left[y_{i}\left(x_{\left(i+1\right)}-x_{i}\right)+\left(\frac{1}{2}\right)\left(y_{\left(i+1\right)}-y_{i}\right)\left(x_{\left(i+1\right)}-x_{i}\right)\right]. \end{array}$$

Consequently FMD was normalized for shear rate (FMD/shear rate) [19].

### 2.4. Supervised Exercise Training

Individuals included in the study habitually exercised at the “Silver Fitness” program going on at the Department of Neurosciences, Biomedicine and Movement Sciences, Section of Movement Sciences, at the University of Verona, Italy. All individuals exercised at least twice a week for 90 minutes combining moderate intensity endurance and resistance training. Sessions usually started with 15 minutes of warm up, which included active joint mobilization and walking on a treadmill or cycling at a preferred speed. Then, individuals performed two 15-minute endurance exercises (either on a cycle ergometer, treadmill, or arm-cranking ergometer) at 70% of the maximal heart rate (calculated using the Karvonen formula: 220 − age in years). Subsequently, individuals performed 3 sets of 8 to 12 reps of resistance exercises at 60–75% of 1 repetition maximum (1RM). 1RM was determined by means of the Brzycki method [22] for all the isotonic ergometers included in the training (i.e., chest press, lat machine, leg press, and others, Technogym, Gambettola, Italy) every year at the beginning of the training program and every 4 months over the year to adjust the work load for the improvements achieved. Exercise sessions ended with stretching exercises for all the muscles involved in the training. All training sessions were supervised by kinesiologists, with a ratio of 1 : 6 (1 kinesiologist supervising 6 participants).

### 2.5. Statistical Analysis

Data are expressed as mean ± SD and minimum-maximum range. One-way ANOVA was used to identify between-group differences for parametric variables (age, systolic blood pressure, FMD%, and shear rate), followed by the Holm-Sidak test. The Kruskal-Wallis one-way analysis of variance on ranks was used for nonparametric variables (quantity of physical activity, BMI, diastolic blood pressure, FMD/shear rate, 6-MWT, CRP, IL-1ra, IL-1*β*, IL-6, IL-8, IL-10, MCP-1, and TNF-*α*), followed by the Tukey post hoc test. The Pearson product moment correlation was used to identify correlations between vascular function variables and inflammation markers. All statistical analyses were performed with SigmaPlot Windows Version 12.0 (Systat Software, Chicago, IL).

## 3. Results

### 3.1. Characteristics of the Participants

Individuals included in this study were 70 ± 5 years old and included 39 females and 31 males. The most common medications taken by the participants were statins, taken by 24% of the participants; diuretics, taken by 17% of the participants; and antiacids, taken by 16% of the participants. Interestingly, although a history of high blood pressure was not reported by the participants, on average both of the groups exhibited high values of systolic blood pressure (see Table 1). We also investigated the sex-specific differences in the variables we assessed; however, differences between males and females were not found. Referring to the quantity of physical activity, on average all individuals included in the study have been taking part in the exercise program for 5 ± 1 years and were involved 3 ± 1 days/week, for a total amount of 270 ± 60 minutes per week (see Table 1).

### 3.2. Lean vs. Overweight and Obese Subjects

**Figure 1:**
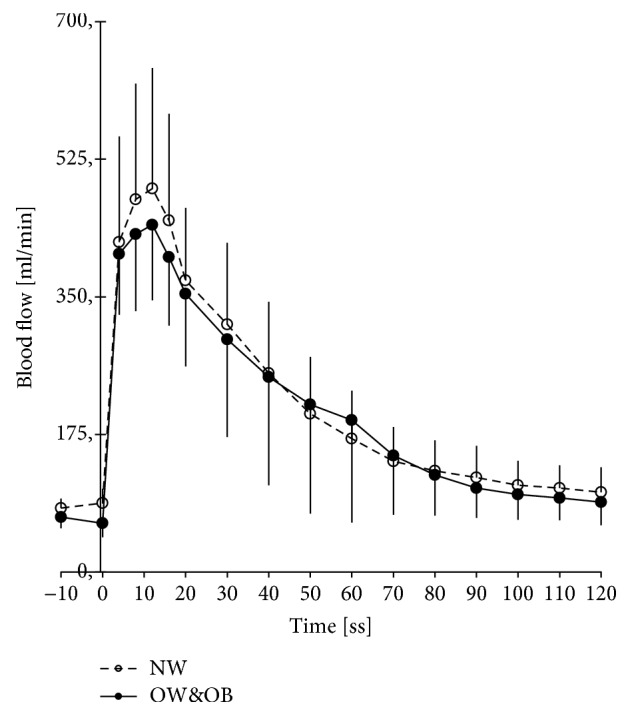
Reactive hyperemic response during the FMD test. Increase in brachial artery blood flow in the normal weight (NW) group and overweight and obese (OW&OB) group during the 2 minutes following a 5-minute supersystolic occlusion during the FMD test.

**Figure 2:**
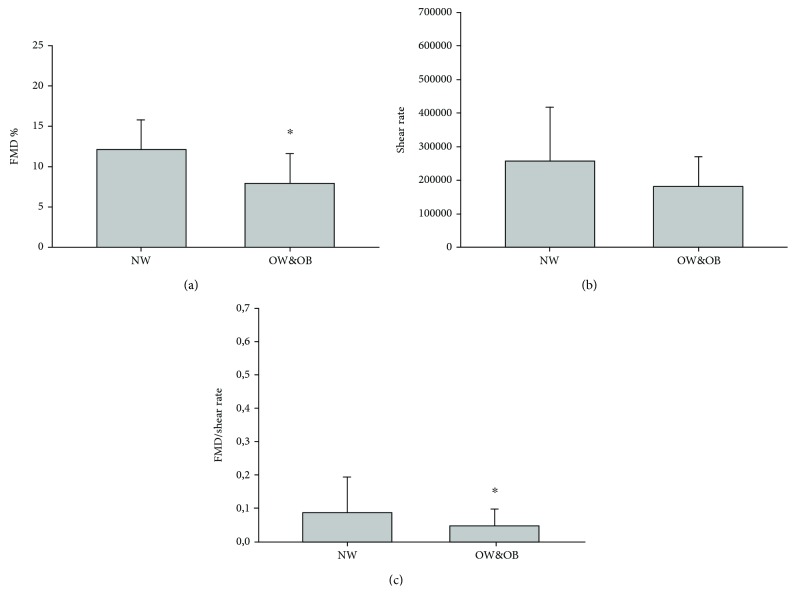
Flow-mediated dilation in normal weight and overweight and obese elderly subjects. ^∗^Statistical difference between groups, *p* < 0.005.

**Figure 3:**
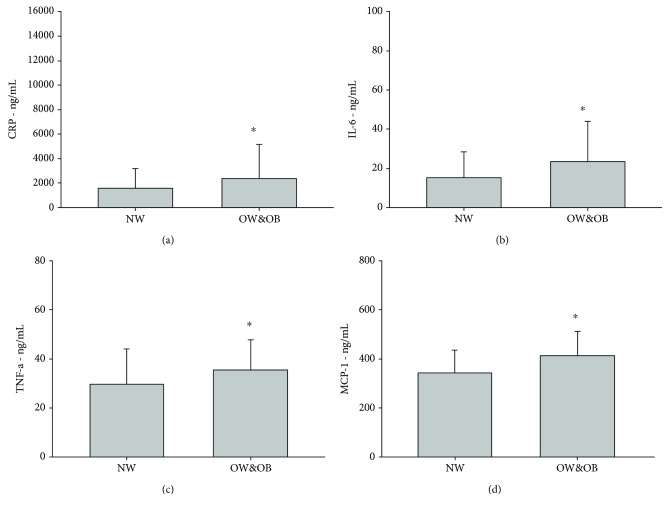
Proinflammatory markers in normal weight and overweight and obese elderly subjects. C-reactive protein (CRP, a); interleukin-6 (IL-6, b); tumor necrosis factor-*α* (TNF-*α*, c); and monocyte chemoattractant protein-1 (MCP-1, d). ^∗^Statistical difference between groups, *p* < 0.005.

NW and OW&OB did not differ for age, number of males and females in the groups, medicine intake, years practicing physical activity, weekly amount of physical activity, and performance measured as 6-MWT (see Table 1). As expected, the OW&OB group had a 20.6% higher BMI compared with the NW group (*p* < 0.001). Regarding the vascular function, no difference between groups was found in the hyperemic response during the FMD test (see Figure 1) or in the shear rate (see Figure 2(b)). Nevertheless, the OW&OB group exhibited a 9.8% higher systolic blood pressure (*p* = 0.001) and an 8.6% higher diastolic blood pressure (*p* = 0.005), as well as a 35.5% lower FMD% (see Figure 2(a), *p* < 0.001) and a 39.7% lower FMD/shear rate (see Figure 2(c), *p* = 0.001) than the NW group. Concerning the inflammatory profile, no significant differences between groups were found for many cytokines, such as IL-10, IL-1ra, IL-1*β*, and IL-8. However, the OW&OB group exhibited higher values of proinflammatory cytokines, such as a 32.9% higher CRP level (see Figure 3(a), *p* = 0.005), a 35.5% higher IL-6 level (see Figure 3(b), *p* = 0.048), a 15.8% higher TNF-*α* level (see Figure 3(c), *p* = 0.0031), and a 16.7% higher MCP-1 level (see Figure 3(d), *p* = 0.004) compared with the NW group. When investigating the correlation between vascular function and inflammatory profile, no significant results were found in NW or OW&OB (see Table 2).

## 4. Discussion

Although several studies have investigated the effect of exercise on age-related and obesity-related morbidities, no studies have aimed at investigating the exercise-induced effects on vascular function and inflammatory profile in age-related obesity without inclusion of caloric restriction. Numerous studies highlighted the fact that during the aging process there is an increase in overall adiposity and a decrement in fat-free mass contributing to lower vascular function and an increase in the low-grade inflammatory profile. Numerous reviews about vascular function and inflammatory profile in obese older adults also recommend exercise as an effective intervention [7, 8, 10]. However, no studies have investigated the effect of exercise without caloric restriction on vascular function and inflammatory profile in older obese adults. In the present study, we measured endothelial function and inflammatory profile in habitually exercising obese and nonobese older adults who have been taking part in a structured, supervised exercise program for several years without caloric restriction. In accordance with our hypothesis, the effects of elevated adiposity on endothelial function and inflammatory profile were not blunted in exercising overweight and obese individuals, demonstrating that exercise alone cannot serve as a fully successful strategy. Moreover, even though obese individuals exhibited poorer vascular function and higher proinflammatory values, these two phenomena do not seem to be correlated.

### 4.1. Evidence about the Efficacy of Exercise on Vascular Functions in Age-Related Obesity

Although several studies have already investigated the effects of exercise intervention on vascular functions in aging and obesity supporting a positive exercise-induced adaptation, the majority of the studies investigated short-term exercise programs administered to previous sedentary obese subjects [4, 6]. Vinet et al. [6] investigated the effect of short-term low-intensity exercise training in middle-age obese men. Ten individuals were recruited and tested for brachial FMD before and after 8 weeks of an individualized low-intensity program, without any dietary intervention. Compared with normal weight controls, obese individuals exhibited poorer FMD; however, exercise training ameliorated FMD values in the obese group. The authors concluded that a short-term low-intensity exercise training improves endothelium-dependent vasodilation in sedentary middle-age obese men [6]. Another study by Dow et al. [4] showed that regular aerobic exercise reduces ET-1-mediated vasoconstrictor tone in overweight and obese middle-aged adults, and they concluded that these mechanisms may be very important in the exercise-induced improvement in endothelium-dependent vasodilator function in this population.

Our results are not in agreement with the mentioned studies. Indeed, the obese elderly individuals included in our study, who were not undertaking a caloric restriction diet, showed a significantly decreased vascular function compared with age-matched counterparts even though the subjects have been exercising regularly for several years. There was also no statistical difference in the amount of physical activity practiced in the two groups. In support of our results, Lind et al. [5] demonstrated that the increased level of self-reported physical activity does not fully eliminate the deleterious cardiovascular consequences associated with overweight and obesity. One reason supporting our findings may reside directly in the physiology of adipose tissue.

Indeed, adipose tissue acts as an endocrine organ that releases bioactive molecules known as adipokines, overexpressed in obese individuals, leading to a proinflammatory status. Among adipose tissue depots, the perivascular adipose tissue (PVAT) displays a unique physiological role that is a paracrine regulation of vascular function. At the physiological level, PVAT exerts regulatory effects on metabolism and inflammatory response via the local release of hormones, cytokines, and reactive oxygen and nitrogen species. When PVAT exceeds the physiological level, it carries an anticontractile property that influences arteriolar responses to agonists contributing to the regulation of blood flow, nutrient uptake, and tissue homeostasis. Moreover, PVAT is susceptible to inflammation and in obesity infiltration of immune cells into the PVAT is aggravated and the amount of PVAT alongside large and small vessels is augmented, exerting anticontractile effects on conduit and resistance arteries [23]. Consequently, an excess of PVAT might contribute to explain how impaired adipose tissue homeostasis affects vascular functions in obese elderly individuals.

### 4.2. Evidence about the Efficacy of Exercise on Inflammatory Profile in Age-Related Obesity

Recent literature started to focus on long-term physical activity and inflammatory biomarkers in obese subjects, but results are still contrasting. Christiansen et al. [16] aimed to investigate the effect of exercise training and diet-induced weight loss alone or in combination with inflammatory biomarkers. By combining the weight loss in all three groups, the authors found a correlation between the degree of weight loss and improvement in several of the inflammatory markers. The authors concluded that the rather large weight losses (<5–7%) were found to have beneficial effects on circulating inflammatory markers in obese subjects. Furthermore, aerobic exercise for 12 weeks was found to have no effects on circulating inflammatory markers in these subjects, suggesting that a more intensive exercise may be necessary to affect systemic inflammation in obese subjects [16]. Brunn et al. [17] aimed to investigate the effect of a 15-week lifestyle intervention (hypocaloric diet and daily exercise) on inflammatory biomarkers in severely obese subjects. The intervention reduced body weight and increased insulin sensitivity. It also increased plasma adiponectin, while it decreased CRP, IL-6, IL-8, and MCP-1 levels. In agreement with the previous study, the authors concluded that the combination of hypocaloric diet and moderate physical activity resulted in a significant general decrease in the level of inflammation [17]. Pischon et al. [24] investigated the relationship between physical activity and the obesity-related inflammatory markers CRP, IL-6, and soluble TNF-receptors (sTNF-Rs) 1 and 2. The authors even examined the relationship between physical activity and insulin sensitivity and whether inflammatory markers mediate this association. In the study, 405 healthy men and 454 healthy women were included and information about physical activity and other variables were assessed by questionnaires. Results showed that physical activity was inversely associated with the plasma levels of sTNF-R1 and 2, IL-6, and CRP, but after having adjusted for BMI and leptin (as a surrogate of fat mass), most of these association were no longer significant [24]. Our results are partially in accordance with the mentioned studies. First, even though obese individuals without a caloric restriction diet included in the study are considered very active, no exercise-induced adaptation on the inflammatory profile is seen. As previously mentioned, although our subjects have been active already for several years and there is no difference in the amount of physical activity between groups, they did not experience great weight changes in the last 5 years, limiting the effects of the regular physical activity they practice. In obesity, an increased fat mass is accompanied by alterations in the cellular composition and physiology of adipose tissue. Hypertrophy and increased distribution of adipocytes, together with inflammation and dysregulated adipokine secretion, characterize a dysfunctional adipose organ which contributed to the development of several obesity-associated morbidities [23]. Furthermore, in obesity the interplay between adipocytes and immune system components changes. Immune cells secrete cytokines that enhance adipose tissue inflammation, and at the same time, adipocytes express classical macrophage features. As the adipose tissue expands, the populations of macrophages, mast cells, B cells, and T cells increase considerably. As a consequence, there is an increased secretion of the inflammatory adipokines TNF-*α* and CRP and a reduction of anti-inflammatory adipokines [23]. In turn, proinflammatory adipokines and CRP might express their action on endothelial cells compromising their proliferative rate and angiogenic potential, which may contribute to the decline of vascular health and development of symptoms such as hypertension [25]. Although exercise can be considered an anti-inflammatory treatment as seen in many studies, it appears that if exercise is not accompanied by an important caloric restriction with consequent weight loss, the effect of exercise on the inflammatory profile is poor. Indeed, only when exercise decreases body fat and adipocyte hypertrophy will the number of inflammatory cells contained within the adipose tissue decline [23]. This could explain the results found in the individuals included in our study. While they are very active, accumulating about 250 min of physical activity a week, this does not seem to be enough to counteract the harmful effect of age-related adiposity on the inflammatory profile of overweight and obese elderly people [24, 26].

### 4.3. Limitations

Although the results of our study are consistent, some limitations have to be highlighted. First, we did not have any additional control group such as a sedentary NW group and a sedentary OW&OB group. These two control groups would have allowed a better understanding of the exercise-induced adaptation in elderly obese individuals who habitually exercise. Second, we did not investigate any difference between the subgroups of overweight and obese who could exhibit different responses. Third, we did not measure any other parameter which could have helped in the description of the subjects and in the interpretation of the results such as lipid profile, hematocrit, and white blood cell analysis.

## 5. Conclusion

Our hypothesis was that regular exercise would not have helped in maintaining a good inflammatory profile and vascular function in overweight and obese elderly individuals. We also hypothesized that poorer vascular function would have been related to higher proinflammatory markers in this population. Although significant differences were found for the measured variables between overweight and obese subjects compared with nonobese elderly individuals, the results of our study demonstrated that regular, supervised exercise without a caloric restriction diet is not a fully successful stimulus able to counteract the deleterious effect of age-related adiposity on vascular function and inflammatory profile. Moreover, contrary to our second hypothesis inflammation and vascular function do not seem to be correlated and further studies are needed in order to understand the mechanisms underlying the exercise-induced physiological processes that may ensure protection against aging and obesity effects, and studies should be focused on an efficient exercise program able to trigger positive physiological mechanisms against age-induced obesity-related morbidities.

## Figures and Tables

**Table 1:** Subjects characteristics and between groups comparison.

	NW (*n* = 30)	OW&OB (*n* = 40)	*p*
Age (years)	70.3 ± 4.7	69.4 ± 5.4	ns
Female/male (*n*)	16/14	23/17	ns
BMI (kg ∙ (m^2^)^−1^)	23.9 ± 2.6	30.1 ± 2.3	*p* < 0.001
Sys (mmHg)	133.1 ± 14.7	147.5 ± 16.2	*p* = 0.001
Dia (mmHg)	76.7 ± 7.6	83.9 ± 10.4	*p* = 0.005
Medications			
Statin—*n* (%)	7 (23)	10 (25)	ns
Diuretics—*n* (%)	5 (17)	7 (18)	ns
Antiacid—*n* (%)	5 (17)	6 (15)	ns
Physical activity			
Years	5 ± 1	5 ± 2	ns
Min/week	209.4 ± 82.5	221.7 ± 71.3	ns
Days/week	3 ± 1	3 ± 1	ns
6MWT (m)	610.1 ± 68.5	610.0 ± 83.7	ns
Inflammatory profile			
IL-10 (ng/mL)	18.9 ± 22.6	16.6 ± 17.2	ns
IL-1ra (ng/mL)	61.9 ± 33.6	52.7 ± 24.1	ns
IL-1*β* (ng/mL)	9.1 ± 7.2	7.8 ± 5.1	ns
IL-8 (ng/mL)	5.9 ± 4.4	7.1 ± 4.1	ns

^∗^Plus-minus values are means ± standard deviation (SD). One-way ANOVA was used to identify between-group differences for parametric variables, followed by the Holm-Sidak test. The Kruskal-Wallis one-way analysis of variance on ranks was used for nonparametric variables, followed by the Tukey test. Note: NW: normal weight; OW&OB: overweight and obese; BMI: body mass index, activity (min/week); Sys: systolic blood pressure; Dia: diastolic blood pressure; FMD: flow-mediated dilation; 6MWT: 6-minute walking test; CRP: C-reactive protein; IL: interleukin; MCP-1: monocyte chemoattractant protein-1; TNF-*α*: tumor necrosis factor-*α*; *p* = *p* value.

**Table 2:** Pearson's correlation between vascular function and inflammatory profile in normal weight and overweight and obese groups.

	CRP	IL-10	IL-1ra	IL-1B	IL-6	IL-8	MCP-1	TNF-*α*
*Normal weight*								
FMD%	0.216	0.372	0.608	0.225	0.974	0.397	0.980	0.402
Shear rate	0.252	0.820	0.867	0.178	0.295	0.458	0.962	0.534
FMD/shear rate	0.679	0.748	0.492	0.651	0.325	0.748	0.385	0.620
*Overweight & obese*								
FMD%	0.237	0.800	0.430	0.383	0.248	0.177	0.121	0.709
Shear rate	0.469	0.718	0.640	0.300	0.666	0.799	0.438	0.297
FMD/shear rate	0.670	0.729	0.755	0.616	0.540	0.464	0.582	0.722

Note: CRP, C-reactive protein (ng/mL); IL, interleukin (ng/mL); MCP-1, monocyte chemoattractant protein-1 (ng/mL); TNF-*α*, tumor necrosis factor-*α* (ng/mL). No statistical correlation between variables in both groups were found.

## Data Availability

The data used to support the findings of this study are available from the corresponding author upon request.

## Abbreviations

NW: Normal weight individuals
OW&OB: Overweight and obese individuals
NO: Nitric oxide
ROS: Reactive oxygen species
BMI: Body mass index
6MWT: 6-Minute walking test
IL-1ra: Interleukin-1ra
IL-1*β*: Interleukin-1*β*
IL-6: Interleukin-6
IL-8: Interleukin-8
IL-10: Interleukin-10
TNF-*α*: Tumor necrosis factor-*α*
MCP-1: Monocyte chemoattractant protein-1
CRP: C-reactive protein
FMD: Flow-mediated dilation
BF: Blood flow
1RM: 1 repetition maximum
ET-1: Endothelin-1
PVAT: Perivascular adipose tissue.
